# Prevalence of Workaholism Among Egyptian Healthcare Workers With Assessment of Its Relation to Quality of Life, Mental Health and Burnout

**DOI:** 10.3389/fpubh.2020.581373

**Published:** 2020-11-26

**Authors:** Zeinab A. Kasemy, Eman E. Abd-Ellatif, Asmaa A. Abdel Latif, Nadia M. Bahgat, Hanaa Mohammad Abo Shereda, Safaa Ibrahim Shattla, Samira E. Aboalizm, Asmaa Hamed Abd Elhy, Abeer R. Allam, Ahmed N. Ramadan, Hemat Mostafa Amer, Naglaa Abdelmawgoud Ahmed, Abobakr A. AlJifri, Mervat M. El Dalatony

**Affiliations:** ^1^Department of Public Health and Community Medicine, Faculty of Medicine, Menoufia University, Shibin Al Kawm, Egypt; ^2^Department of Public Health and Community Medicine, Faculty of Medicine, Mansoura University, Mansoura, Egypt; ^3^Industrial Medicine and Occupational Health Division of Public Health and Community Medicine Department, Menoufia Faculty of Medicine, Shibin Al Kawm, Egypt; ^4^Department of Anesthesiology and Intensive Care, Faculty of Medicine, Menoufia University, Shebeen El-Kom, Egypt; ^5^Department of Psychiatric and Mental Health Nursing, Faculty of Nursing, Menoufia University, Shibin Al Kawm, Egypt; ^6^Department of Medical Surgical Nursing, Faculty of Nursing, Menoufia University, Shibin Al Kawm, Egypt; ^7^Department of Neuropsychiatry, Faculty of Medicine, Menoufia University, Shibin Al Kawm, Egypt; ^8^Department of Family and Community Health Nursing, Faculty of Nursing, Menoufia University, Shibin Al Kawm, Egypt; ^9^Department of Quality and Patient Safety in Healthcare, Private Hospital Riyadh, Jordan University of Science and Technology, Irbid, Jordan

**Keywords:** burnout, healthcare workers, mental health, pro-inflammatory markers, quality of life, workaholism, work addiction

## Abstract

**Introduction:** Work is a social double edged weapon activity that may have positive and negative effects on individual's quality of life and health.

**Objectives:** To estimate workaholism prevalence and to determine its effects on quality of life, mental health, and burnout among healthcare workers (HCWs).

**Methods:** Using a cross-sectional study, 1,080 Egyptian participants distributed as HCWs and non-HCWs were recruited. The study included 4 questionnaires to assess workaholism, quality of life (QoL), Psychological capital questionnaire (**PCQ**), and General health questionnaire (GHQ). Maslach Burnout Inventory (MBI) was applied to critical specialty HCWs in addition to pro-inflammatory markers including Il6, TNFα, and CoQ10.

**Results:** This study revealed that 24.4 and 24.8% of HCWs were workaholic and hardworking, respectively, in comparison to 5.9 and 28.1% among non-HCWs (*P* < 0.001). Somatic symptoms and anxiety/ insomnia domains of GHQ were higher among HCWs than non-HCWs (*P* < 0.001 and 0.002, respectively). QoL was significantly lower among HCWs than non-HCWs (*P* < 0.001). Workaholism was reported among 43.2% of HCWs with critical specialty (*P* < 0.001). Components of PCQ components were significantly higher among HCWs with critical specialty than non-critical HCWs while QoL showed the reverse (*P* < 0.05). Working excessively was a predictor to burnout [Emotional exhaustion (β = –0.23) and depersonalization (β = −0.25)] and TNFα (β = 0.41). Emotional exhaustion was a predictor to Il6 (β = 0.66), TNFα (β = 0.73), and CoQ10 (β = −0.78).

**Conclusion:** There is a significant association between workaholism and psychologically poor-health and poor quality of life among HCWs. Critical specialty healthcare workers showed association between workaholism, burnout and pro-inflammatory markers. Addressing of personal characteristics, supporting factors in the work environment and periodic examination of the healthcare workers and responding accordingly is required.

## Introduction

Work is a social double edged weapon activity that may have positive and negative effects on individual's life and health (1). In the 1970s, it was the first time to introduce the term “Workaholism” describing a constant need to work which can harm health physically, mentally, psychologically and socially (2, 3). Andreassen et al. (4) defines workaholism (Work addiction) as becoming too anxious, strongly motivated by work, and spending huge efforts into your work leading to social, physical, psychological health impairment. Workaholism has a set of two characteristics occurring together: Working excessively (WE) (behavioral component) and working compulsively (WC) (cognitive component) (5).

In situations that require higher efforts to attain the expected level of performance, job demands might become stressors affecting the entire human life (6). Quality of life (QoL) is how a person perceives his place in life in a frame of culture affecting his objectives, expectations, standards and fears (7). QoL could be affected by many factors like the individual's physical and psychological status, personal beliefs, social relations, and its related prominent environmental features. Epidemiological studies highlighted the negative impacts of overworking on the risks of cardiovascular disorders, fatigue, stress, depression, anxiety, sleeping quality, alcoholism and smoking, mental health status, hypertension, musculoskeletal disorders, and health behaviors in addition to poor quality of life (8–11).

Healthcare providers of different ages are usually highly concerned with their jobs leading to workaholism (12). They are more susceptible to stress and burn-out which is physical, emotional, mental, and spiritual exhaustion as they are in charge of human lives (13, 14). Some researchers suppose that burnout syndrome is a variant of depression (15).

There is strong evidence that depression is accompanied by an increase of proinflammatory cytokines e.g., interleukin-1 (IL-1), IL-2, IL-6, tumor necrosis factor-α (TNFα), and acute phase proteins (16). Coenzyme Q10 (CoQ10) is an antioxidant that has anti-inflammatory effects and lower levels of CoQ10 have been associated with depression in patients (17). Much of the interest in inflammation and depression has been focused on cytokines, which mediate the innate immune response, including tumor necrosis factor alpha (TNF-α), and IL-6, which appears to be one of the most reliable peripheral biomarkers in major depression (18).

Developing an environment promoting resilience for HCWs limits negative, and elevates positive, outcomes of stress in healthcare settings. Optimism is needed for HCWs to see the world as a positive place, accepting and handling difficulties as a challenge not a barrier and as a consequence this will improve their functioning level, patients' satisfaction, and therapeutic outcome.

According to authors' knowledge, no published articles reported the percentage of workaholism among Egyptian healthcare workers. This work aimed to 1- Estimate workaholism prevalence and to determine its effects on quality of life and mental health among healthcare workers (HCWs) 2- Study the relation between workaholism and burnout and their association to pro-inflammatory markers including IL6, TNFα, and Coenzyme Q10 among critical specialty healthcare workers.

### Methods

Using a cross-sectional study, 1,080 participants distributed as HCWs and non-HCWs were recruited. Self-administered questionnaires were used by a team of trained personnel. The trained team members chose the non-crowding days of work and time of rest of HCWs to encourage them to share and to avoid any extra load on them. HCWS of four different hospitals were interviewed. HCWs included physicians and nurses with critical (departments of Surgery, Anesthesia and ICU) and non-critical specialties. For the non-HCWs group, the trained personnel directed their efforts to the administrative board in the involved hospitals of the HCWs and 3 governmental and 2 private sectors to gain wide sharing. The questionnaires were left to be filled in for HCWs and then collected later at an appropriate time while the non-HCWs the trained team collected the data immediately by an interviewer based questionnaire.

### Exclusion Criteria

Any person refused to share or wanted to withdraw at any point of the research was excluded from the study.

### Sample Size

As the frequency of workaholism among Egyptians healthcare workers (HCWs) was not studied before so the occurrence probability equaled to its non-occurrence (p =q = 0.5) and with acceptable limit of precision (D) of 0.03 value, the sample size was estimated to be 1,014 participants divided into 2 groups; HCWs and non-HCWs. To avoid drop out, the sample has increased to 1,126. About 1,126 questionnaires were delivered to both groups in equal numbers. HCWs responded to 540 questionnaires out of 563 ones with a response rate of 95.9%. Non-HCWs group responded to 544 questionnaires with a response rate of 96.6%. Four incomplete questionnaires were excluded leaving 540 ones.

### Tools

The study included 4 questionnaires to assess workaholism, quality of life, Psychological capital components, and General health questionnaire:

- **The adapted version of the Dutch Workaholism Scale (DUWAS)** (19): It contains 10 items of a 4-point scale with a range from 1–4 (Almost never to always); the questionnaire included two equally divided scales: working excessively (WE) and working compulsively (WC). The cutoff point was 2.5 in both scales. More than 2.5 in both scales (WE and WC) equaled workaholic. Above 2.5 (WE) and below 2.5 (WC) equaled hard worker. Below 2.5 (WE) and above 2.5 (WC) equaled compulsive worker. Less than 2.5 in both of (WE and WC) equaled relaxed worker were defined as (5, 20, 21).
- **World Health Organization Quality-of-Life Scale (WHOQOL-BREF)** **(****22****):** a twenty six item revised version was used. It fits most of geographical, ethnic background, and cultural aspects. It consists of 4 categories; physical (*R* = 7–35), mental (*R* = 6–30), social(*R* = 3–15), and environmental (*R* = 8–40) well-being over the past 4 weeks. Low score means Low QOL. The range of reliability test in WHOQOL-BREF is 0.70–0.80.
- **Psychological capital Questionnare (PCQ-12):** (Short form) of the Psychological Capital Questionnaire (PCQ-24). It includes four subscales titled self-efficacy (item numbers 1–4), hope (item numbers: 5–7), resilience (item numbers 8–10), and optimism (item numbers 11–12). A Likert 6 point scale ranging from 1: Strongly disagree, to 6: Strongly agree was used (23–25).
- **General Health questionnaire (GHQ**-28): Four subscales were used to measure the general health state: somatic symptoms, anxiety/insomnia, social dysfunction and symptoms of depression. To rate recent psychological state, a Likert 4 point scale ranging from (1: Not at all, to 4: Much more than usual) was used. Low score means good psychological status (26).

## Healthcare Workers Who Were Working in Critical Specialty (Departments of Surgery, Anesthesia, and ICU) Were Studied Regarding

1. Burnout using **the Maslach Burnout Inventory (MBI)** which is a Likert scale questionnaire, the answer of each question ranged from never = 0 to everyday = 6. It contains 22 items divided into three subscales: emotional exhaustion (EE 9 items, *R* = 0–≥27) depersonalization (DP 5 items, *R* = 0–≥13) and personal accomplishment (PA 8 items, *R* = ≥ 37–0) (27).
2. Blood samples were collected after 8 h of fasting at the early morning. Blood samples were taken by sterile syringe from each participate. 3–4 cm venous blood collected in plan tube and centrifugated. Finally supernatants were removed and stored at −80 °C until determination via ELISA kits. These investigations were used for assessing biomarkers of depression including Interleukin 6 (IL-6) (ELISA KIT‘, Sino Gene Clon Biotech Co.,Ltd, Detection range 4.7 ng/L −800 ng/L), Tumor necrosis factor alpha, TNF-α (ELISA KIT‘, Sino Gene Clon Biotech Co.,Ltd, Detection range 15.6 ng/L −1,000 ng/L) and Coenzyme Q10 (CoQ10) (ELISA Kit, Sino Gene Clon Biotech Co.,Ltd, Detection range: 1.56 ng/ml −50 ng/ml).

### Ethical Approval

Approval of the local institutional research board was obtained.

### Statistical Analysis

SPSS version 22(SPSS Inc., Chicago, IL, USA) was used to analyze the data. Qualitative data were expressed as no and %, while quantitative data were expressed as mean ±SD. Test of normality was conducted. Non-paired *t-*test was used to compare between 2 means of normally distributed variables while ANOVA test was used to compare between means of more than 2 groups for normally distributed variables. For analysis of non-parametric data, Mann-Whitney was used between 2 groups while a Kruskal-Wallis test was employed to compare between more than 2 groups. Qualitative variables were analyzed by Chi-Squared (χ^2^). A Pearson correlation test was used to assess direction and strength of association between quantitative variables. Multiple regression analysis using pathway analysis was applied to detect the predictors between dependent and independent variables. Significance level was set at *P* < 0.05.

## Results

**Figure 1:**
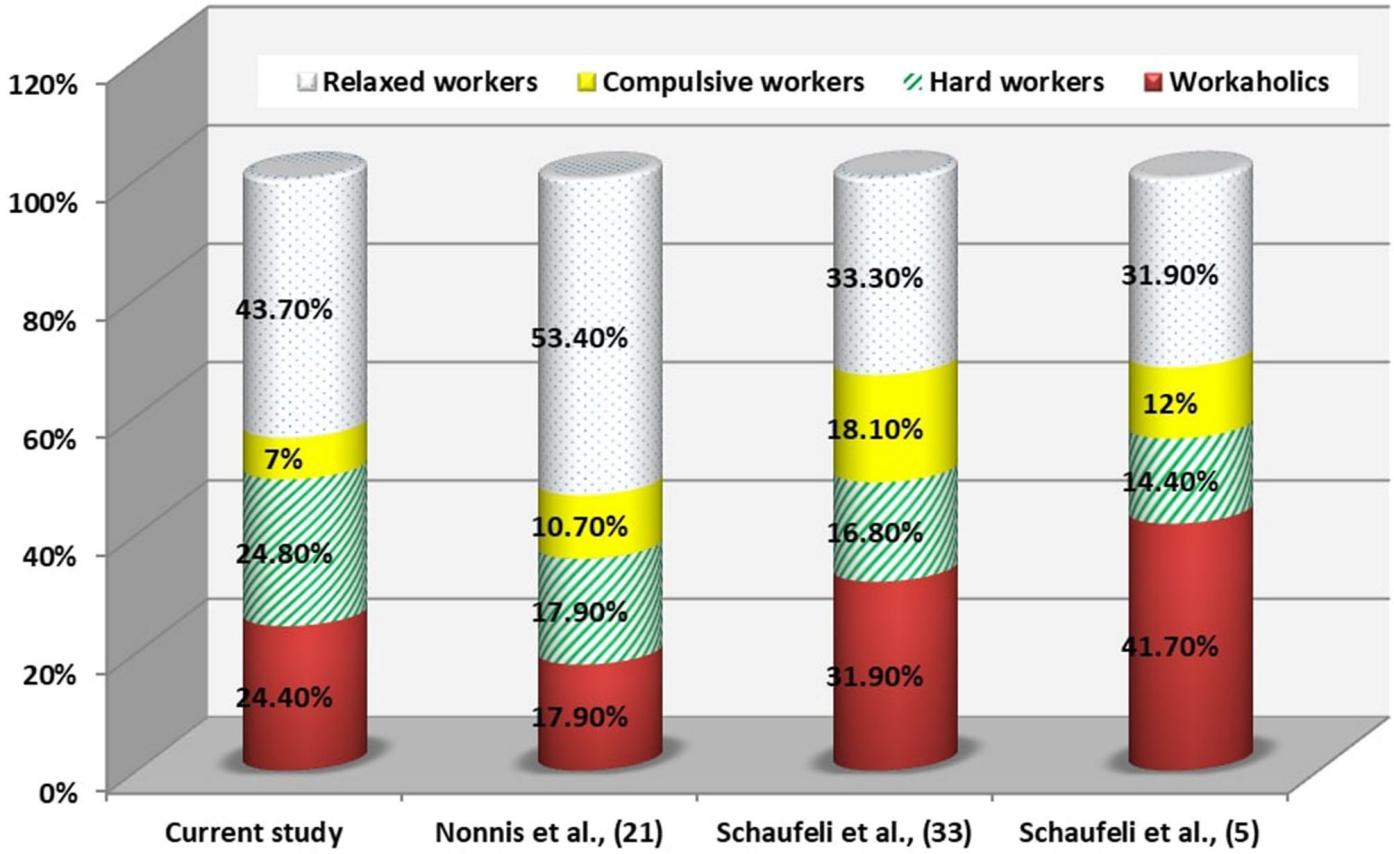
Comparison of the profiles of the current study with other research in health care.

Workaholism was reported in 24% of HCWs and while it was 17.9, 31.9, and 41.7% in Nonnis et al. (21), Schaufeli et al. (33), and Schaufeli et al. (5), respectively (*P* < 0.001) (Figure 1).

**Figure 2:**
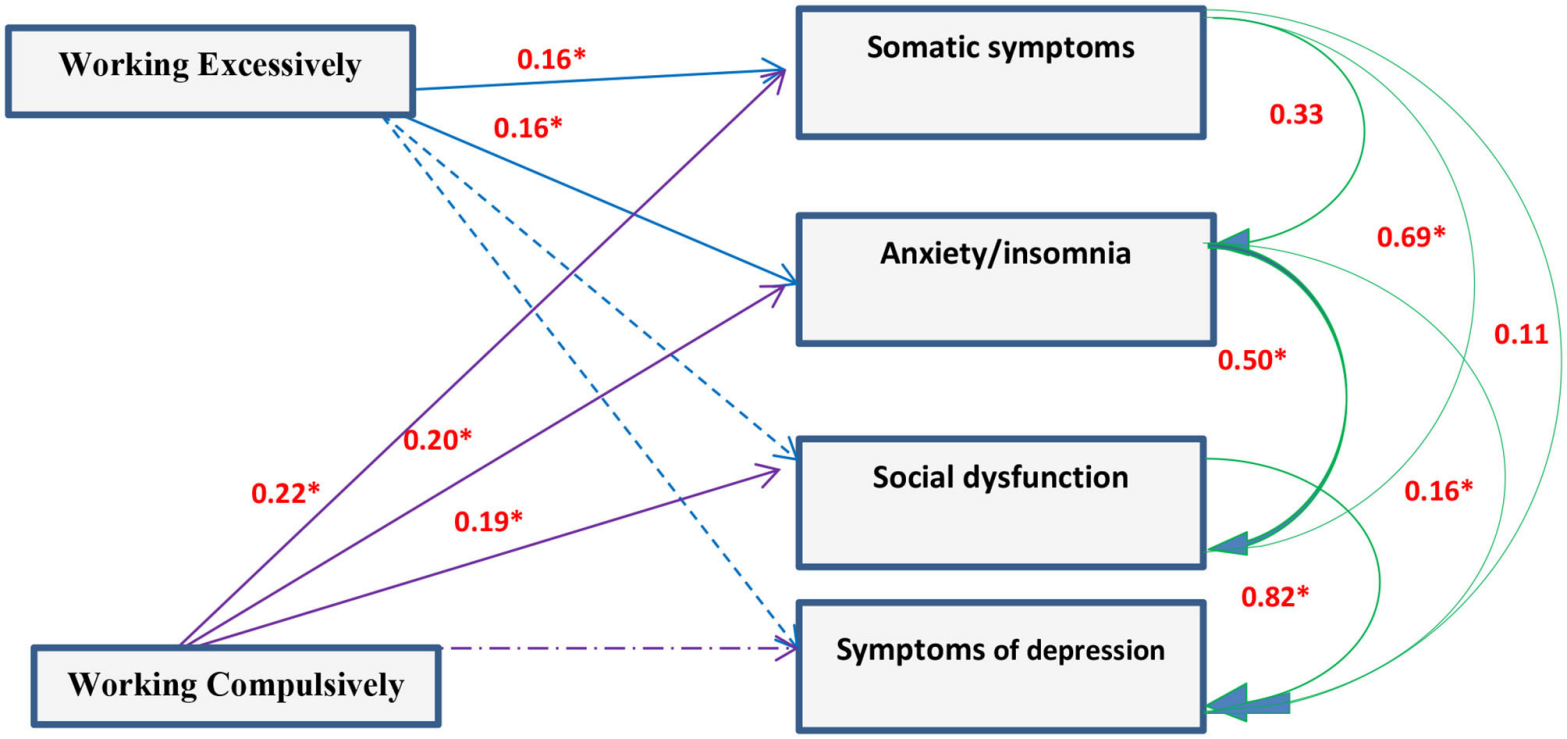
Path diagram of the model used for the whole group of subjects for work addiction scores and GHQ (*n* = 1,080) (Multiple regression analysis using pathway analysis was applied). *significant.

**Figure 3:**
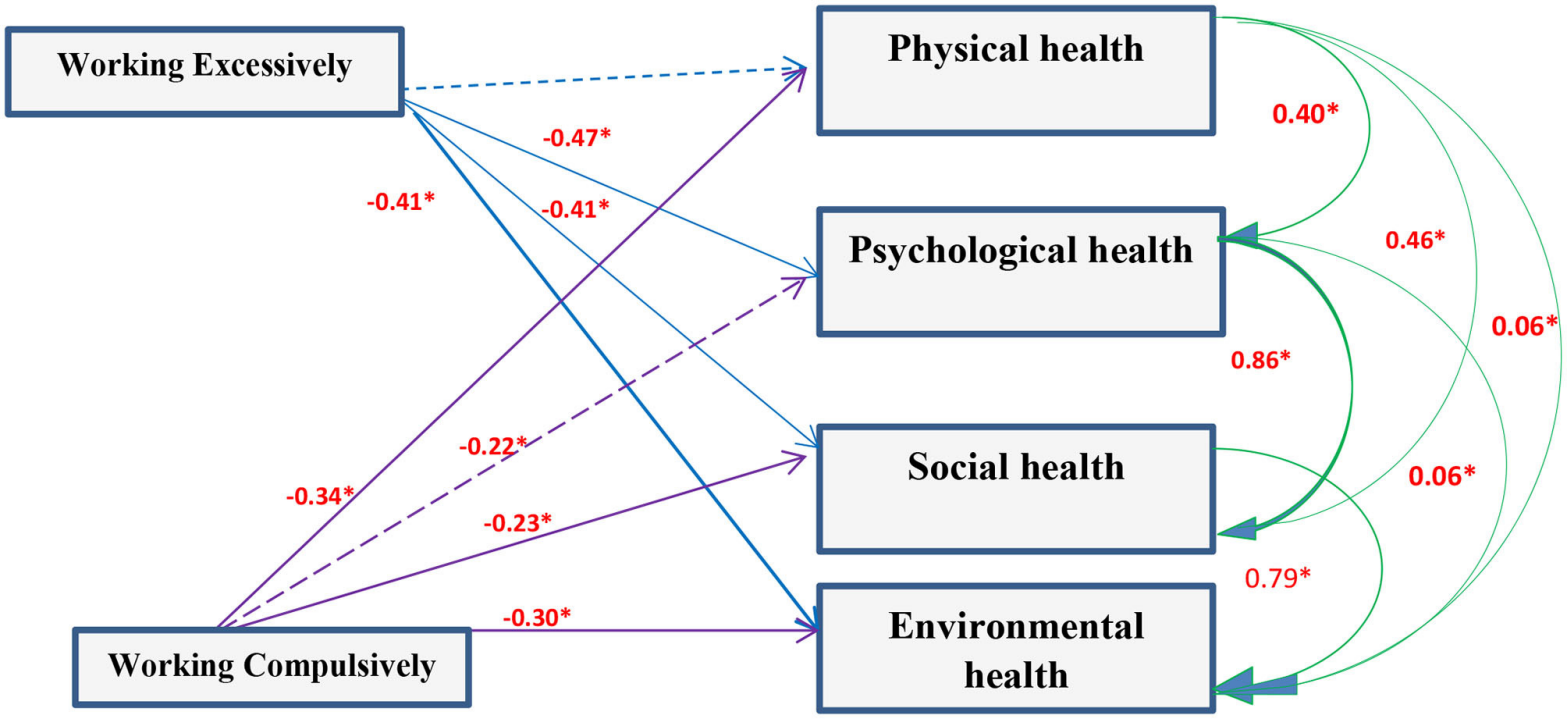
Path diagram of the model used for the whole group of subjects for work addiction scores and Quality of life (*n* = 1,080) (Multiple regression analysis using pathway analysis was applied). *significant.

**Figure 4:**
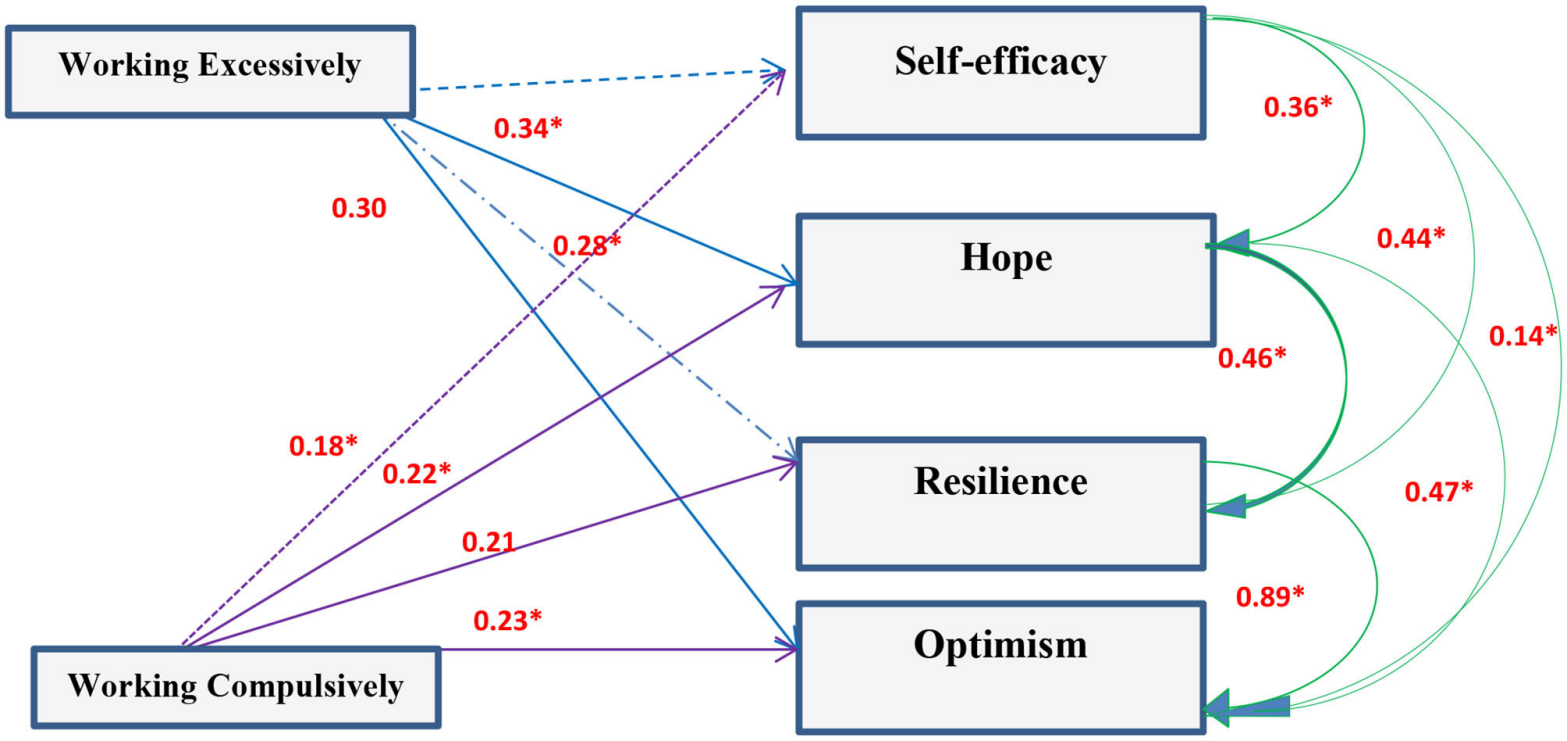
Path diagram of the model used for the whole group of subjects for work addiction scores and Psychological capital components (*n* = 1,080) (Multiple regression analysis using pathway analysis was applied). *significant.

Correlation analysis between scores of the entire studied group working excessively or compulsively revealed that there was a positive correlation with GHQ and **PCQ** items while there was a negative correlation with the quality of life (*P* < 0.05). Working excessively and compulsively were not predictors to GHQ but strong predictors to quality of life and PCQ components (Figures 2–4).

**Figure 5:**
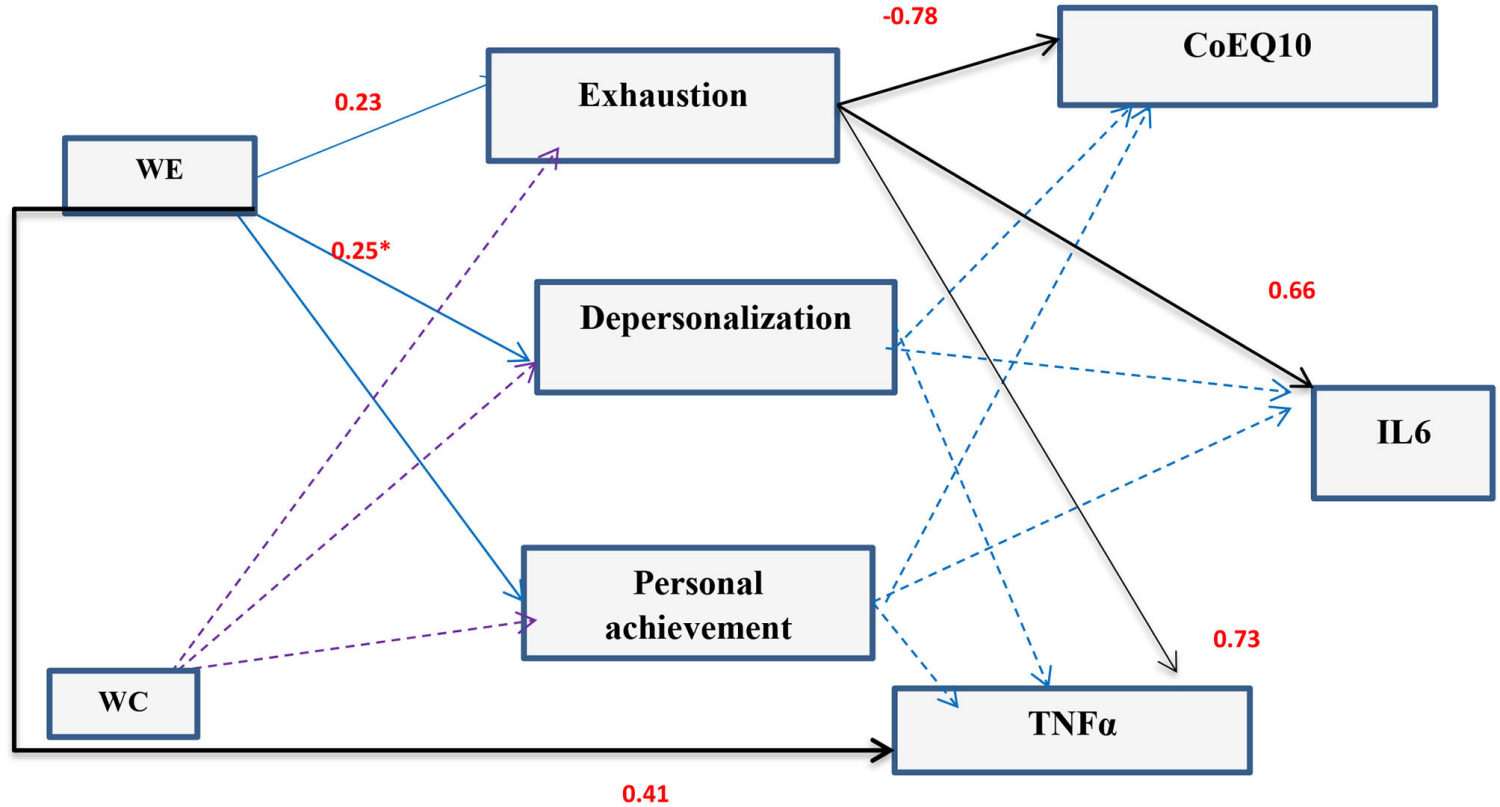
Path diagram of the model used for the whole critical specialty HCWs group for work addiction scores and burnout and pro-inflammatory markers (*n* = 81) (Multiple regression analysis using pathway analysis was applied). *significant.

Working excessively was a predictor to burnout (emotional exhaustion (β = −0.23, CI 95%: 0.03–1.60) and depersonalization (β = −0.25, CI 95%: 0.05–0.94) and TNFα (β = 0.41, CT 95%: 8.73–26.84). Emotional exhaustion was a predictor to Il6 (β = 0.66, CI 95%: 4.25–8.08), TNFα (β = 0.73, CI 95%: 6.64–11.36), and CoQ10 [β = −0.78, CI 95%: −0.55–(−0.35)] (Figure 5).

Working >48 h/week was highly prevalent among HCWs (54.1%) in comparison to non-HCWs (36.3%) and about 73.3% HCWs don't take a weekend. The study revealed that 24.4% of HCWs were workaholic and 24.8% were hardworking in comparison to 5.9 and 28.1%, respectively, among non-HCWs (*P* < 0.001). GHQ results were higher among HCWs than among non-HCWs especially the somatic symptoms and anxiety/ insomnia (*P* < 0.001 and 0.002, respectively). HCWs had significantly lower quality of life than non-HCWs (*P* < 0.001) but there was no significant difference regarding **PCQ components** (*P* > 0.05). The comparison between physicians and nurses revealed that 86.8% of physicians don't take a weekend in comparison to nurses (23.5%). While 2.8% of the physician were relaxed workers, 64.9% of the nurses were relaxed workers (*P* < 0.001). GHQ results were higher and that of QOL were lower among physician than nurses (*P* < 0.001). Regarding **PCQ components**, all of them were significantly higher among physicians than nurses (*P* < 0.001). When studying the specialty of HCWS, it was found that critical specialty had significantly higher working hours and no weekends in comparison to non-critical specialty. Also QOL was significantly lower while PCQ components were significantly higher among HCWs with critical specialty (Table 1).

**Table 1:** Distribution of Dutch Workaholism Scale (DUWAS), General health questionnaire (GHQ), Quality of life (QOL), and Psychological capital components (PCQ) regarding occupation.

	**Occupation**				***P-*value**	**HCWs**				***P-*value**	**Specialty**				***P-*value**
	**HCWs**		**Non-HCWs**			**Physicians**		**Nurses**			**Critical**		**Non-critical**		
	**Mean** **±** **SD**		**Mean** **±** **SD**			**Mean** **±** **SD**		**Mean** **±** **SD**			**Mean** **±** **SD**		**Mean** **±** **SD**		
**Age (years)**	35.60 ± 10.34		36.04 ± 11.25		0.505	36.44 ± 10.21		34.71 ± 10.43		0.052	35.34 ± 10.14		35.64 ± 10.39		0.808
	**No**	**%**	**No**	**%**		**No**	**%**	**No**	**%**		**No**	**%**	**No**	**%**	
**Working hours/week**															
**>48**	292	54.1	196	36.3	<0.001^*^	236	86.8	63	23.5	<0.001^*^	56	69.1	243	52.9	0.007^*^
**≤48**	248	45.9	344	63.7		36	13.2	205	76.5		25	30.9	216	47.1	
**Having a Weekend**	396	73.3	252	46.7	<0.001^*^	182	66.9	276	79.9	0.001^*^	37	45.7	359	78.2	<0.001^*^
**DUWAS**															
Workaholic	132	24.4	32	5.9		100	36.8	32	11.9		35	43.2	97	21.1	
Hard worker	134	24.8	152	28.1	<0.001^*^	92	33.3	42	15.7	<0.001^*^	18	22.2	116	25.3	<0.001^*^
Compulsive worker	38	7.0	44	8.1		18	6.6	20	7.5		2	2.5	36	7.8	
Relaxed workers	236	43.7	312	57.8		62	2.8	174	64.9		26	32.1	210	45.7	
**GHQ**															
Somatic	22.70 ± 5.02		21.59 ± 4.43		<0.001^*^	23.42 ± 5.57		21.97 ± 4.29		0.001^*^	23.38 ± 5.02		22.58 ± 2.02		0.190
symptoms	6.73 ± 1.87		6.22 ± 1.65		<0.001^*^	6.96 ± 1.99		6.50 ± 1.72		0.005^*^	6.97 ± 1.90		6.69 ± 1.86		0.215
Anxiety/insomnia	7.14 ± 1.59		6.85 ± 1.35		0.002^*^	7.34 ± 1.72		6.94 ± 1.43		0.004^*^	7.23 ± 1.43		7.15 ± 1.43		0.040^*^
Social dysfunction	7.17 ± 1.43		7.02 ± 1.16		0.077	7.34 ± 1.56		6.98 ± 1.25		0.003^*^	1.69 ± 1.03		1.64 ± 1.09		0.662^*^
Symptoms of depression	1.65 ± 1.08		1.47 ± 0.74		0.256	1.77 ± 1.21		1.53 ± 0.93		0.065	1.77 ± 1.21		1.53 ± 0.93		0.471
**QOL**															
Physical	64.96 ± 2.64		66.01 ± 2.37		<0.001^*^	64.02 ± 2.41		65.92 ± 2.53		<0.001^*^	63.98 ± 2.65		65.14 ± 2.60		<0.001^*^
Mental	64.37 ± 2.56		65.17 ± 2.54		<0.001^*^	63.44 ± 2.26		65.31 ± 2.52		<0.001^*^	63.62 ± 2.56		64.50 ± 2.55		0.005^*^
Social	60.0 ± 6.94		62.29 ± 6.97		<0.001^*^	57.61 ± 6.14		62.42 ± 6.88		<0.001^*^	58.06 ± 6.63		60.34 ± 6.94		0.005^*^
Environmental	55.67 ± 10.64		60.12 ± 10.26		<0.001^*^	51.73 ± 9.39		59.66 ± 10.36		<0.001^*^	53.60 ± 10.75		56.03 ± 10.59		0.058
**PCQ**															
Elf-efficacy	16.98 ± 3.26		16.96 ± 3.06		0.923	18.23 ± 2.71		15.71 ± 3.29		<0.001^*^	17.82 ± 3.17		16.83 ± 3.26		0.010^*^
Hope	11.11 ± 3.70		10.82 ± 3.33		0.017^*^	12.66 ± 3.09		9.53 ± 3.59		<0.001^*^	12.17 ± 3.58		10.92 ± 3.69		0.005^*^
Resilience	9.16 ± 3.16		9.14 ± 2.95		0.467	10.47 ± 2.59		7.82 ± 3.13		<0.001^*^	10.01 ± 3.03		9.0 ± 3.16		0.007^*^
Optimism	4.73 ± 2.61		4.78 ± 2.42		0.608	5.61 ± 2.58		3.59 ± 2.60		<0.001^*^	5.33 ± 2.64		4.62 ± 2.60		0.012^*^

* *Significant, Non-paired t and ANOVA tests were used for normally distributed variables. For analysis of non-parametric data, Mann-Whitney, and Kruskal-Wallis tests were employed. Qualitative variables were analyzed by Chi-Squared (χ^2^)*.

The young age groups showed more working hours than old age ones (*P* < 0.001). Workaholism and working hard were reported among 20.7% among young ages in comparison to 8% among old one (*P* < 0.001). Results of GHQ and **PCQ** components were significantly higher among young ages than old ones while QOL showed the reverse (*P* < 0.001). Males are working more hours than females. Males were workaholic (17.7%) in comparison to 11.9% among females. Results of GHQ and **PCQ** components were higher among males than females while QOL showed the reverse (Table 2).

**Table 2:** Distribution of Distribution of Dutch Workaholism Scale (DUWAS), General health questionnaire (GHQ), Quality of life (QOL), and Psychological capital questionnaire (PCQ) regarding age and sex of the studied group.

	**Age (years)**				***P-*value**	**Sex**				***P-*value**
	**>40**		**≤40**			**Male**		**Female**		
	**No**	**%**	**No**	**%**		**No**	**%**	**No**	**%**	
**Working hours/week**										
>48	157	33.8	338	55.0		303	49.7	192	40.9	
≤48	308	66.2	277	45.0	<0.001^*^	307	50.3	278	59.1	0.004^*^
**Having a Weekend:**	287	61.7	361	58.7	0.316	372	61.0	276	58.7	0.452
**DUWAS**										
Workaholic	37	8.0	127	20.7		108	17.7	56	11.9	
Hard worker	96	20.6	190	30.9	<0.001^*^	174	28.5	112	23.8	<0.001^*^
Compulsive worker	16	3.4	66	10.7		66	10.8	16	3.4	
Relaxed workers	316	68.0	232	37.7		262	43.0	286	60.9	
	**Mean** **±** **SD**		**Mean** **±** **SD**			**Mean** **±** **SD**		**Mean** **±** **SD**		
**GHQ**	21.64 ± 4.45		22.53 ± 4.96		0.003^*^	22.62 ± 5.06		21.53 ± 4.28		<0.001^*^
Somatic symptoms	6.29 ± 1.67		6.62 ± 1.85		0.003^*^	6.64 ± 1.88		6.27 ± 1.62		0.001^*^
Anxiety/insomnia	6.79 ± 1.36		7.15 ± 1.55		<0.001^*^	7.18 ± 1.52		6.77 ± 1.41		<0.001^*^
Social dysfunction	7.01 ± 1.21		7.16 ± 1.36		0.070	7.16 ± 1.37		7.01 ± 1.21		0.063
Symptoms of depression	1.53 ± 0.89		1.58 ± 0.96		0.397	1.63 ± 1.0		1.48 ± 0.83		0.006^*^
**QOL**										
Physical	66.13 ± 2.47		65.0 ± 2.53		<0.001^*^	65.16 ± 2.46		65.91 ± 2.62		<0.001^*^
Mental	65.60 ± 2.40		64.15 ± 2.55		<0.001^*^	64.44 ± 2.50		65.20 ± 2.64		<0.001^*^
Social	63.02 ± 6.87		59.73 ± 6.86		<0.001^*^	60.25 ± 6.83		62.31 ± 7.17		<0.001^*^
Environmental	60.35 ± 10.62		56.04 ± 10.36		<0.001^*^	56.32 ± 10.39		59.92 ± 10.73		<0.001^*^
**PCQ**										
Self-efficacy	15.97 ± 3.31		17.72 ± 2.82		<0.001^*^	17.28 ± 3.09		16.56 ± 3.21		<0.001^*^
Hope	9.90 ± 3.59		11.77 ± 3.25		<0.001^*^	11.36 ± 3.48		10.45 ± 3.51		<0.001^*^
Resilience	8.16 ± 3.12		9.89 ± 2.79		<0.001^*^	9.45 ± 3.01		8.75 ± 3.07		<0.001^*^
Optimism	3.93 ± 2.56		5.38 ± 2.29		<0.001^*^	5.02 ± 2.48		4.40 ± 2.52		<0.001^*^

* *Significant, ANOVA test was used for normally distributed variables. Kruskal-Wallis test was employed. Qualitative variables were analyzed by Chi-Squared (χ^2^)*.

## Discussion

Workaholism seems to be challenge functioning as a resource and a demand for healthcare workers (HCWs) (28). The potential to energize workers makes workaholism able to create pleasure in work, hence more job satisfaction with positive emotional effects (29). A health impairment psychological process and feeling of emotional exhaustion among HCWs may be achieved at higher levels of workaholism causing a so-called “loss spiral” and hence job dissatisfaction (29–32).

### Prevalence of Workaholosm

In the current study, workaholism among HCWs represented 24%. It is lower than that of Schaufeli et al. (33) and Schaufeli et al. (5) In the Netherlands and Japan which was 41.7 and 31.9%, respectively. Hu et al. (34) explained this variation by the tendency of employees in the Western cultures to be more work engaged than their peers in the Eastern cultures. But the current results were higher than that of Nonnis et al. (21) in Italy which was 17.9%. There are many factors could affect workaholism as gender, personality, cultural values within the countries but regardless of cultures, workaholism is injurious to the employees' well-being. Work addiction could be explained by the compulsive need to achieve status and success or to escape emotional stress. Hard work and diligence are known as desirable traits among physicians. It is common among people described as perfectionists who are at risk of the belief that the best is never good enough and this is concerning as HCWs' well-being is essential to ensure high quality healthcare services (35). Prevalence of workaholism among non-HCWs was 5.9% in the current results which is lower than a study carried out in Norway (4). The prevalence rates of workaholism ranging from 0.3 to 46.6% depending of the cut-off employed. In the present study an endorsement of at least 2.5 as the cut-off was used, and is in line with the authors' previous suggestion (20, 21).

### Relation Between Workaholism, Mental Health, and Quality of Life

Analysis of the correlation between scores of the entire studied group and working excessively or compulsively showed that there was positive correlation with GHQ and Psychological capital items while there was negative correlation with quality of life. There is an association between workaholism and ill-health whether psychologically or physically (5, 36). Working compulsively has a cognitive pattern making individuals work hard leading to physical or mental exhaustion (29, 37). Hard work and diligence are known as desirable traits among physicians. Developing an environment promoting resilience for HCWs limits negative, and elevates positive, outcomes of stress in healthcare settings. Optimism is needed for HCWs to see the world as a positive place, accepting and handling difficulties as a challenge not a barrier and as a consequence this will improve their functioning level, patients' satisfaction, and therapeutic outcome. So working hard is associated with strong hope, resilience, and optimism (38–40).

The present findings reported that working excessively is more likely to be associated with psychophysical exhaustion and this agrees with Delgado et al. (35) and Andreassen et al. (41). Physical, mental, social, and environmental domains of quality of life were significantly different between HCWs and non-HCWs and also between physicians and nurses. Workaholism is associated with better health through active coping, and with poor health through emotional discharge.

The present association between workaholism and psychological ill health agrees with many studies (20, 42–45). There were great similarities between participants working excessively and compulsively regarding psychological distress, psychosomatic symptoms, job stress and impaired well-being (20, 46–48). On the base of above/below dichotomy Buelens and Poelmans (28), compared 8 groups of workers regarding work involvement, drive, and work enjoyment (3 workaholism dimensions). Work addicts had high scores in work involvement and drive, and low scores on enjoyment in addition to unfavorable scores on work-to-family conflict, work conflict, satisfaction with family and colleagues, and stress and health complaints in comparison with relaxed workers. Schaufeli et al. (33) found that engaged peers of workaholic managers were more satisfied with their jobs and less psychologically distressed. Dissimilarities between excessive and compulsive workers lie in perfectionism sought by the compulsive working group (49). Porter (50) explained poor social relationships at work by anger and frustration characterizing perfectionist workaholics and this could explain the relatively low positive correlation with PCQ among the working compulsively group in contrast to the working excessively one.

The current results revealed that across several occupations there is an association between workaholism and psychological health. In the same context, positive correlations were found between workaholism, and work overload and number of working hours (20, 47). Critical specialty seems to suffer from workaholism and its associated low QOL and high PCQ components. Especially when enjoying their work, participants experiencing great professional demands, may find difficulty in setting limits for themselves. So to reduce this risk and ensure maintain of a good work-life balance, managers should build supportive environment to all employees (51, 52).

Workaholics tend to have long working hours interfering with family and personal lives. HCWs suffer mental demands (large quantities of information with complicated decisions), organizational demands (working in a complex organizational environment), and emotional demands (dealing with suffering patients and their families). In addition, some demographic variables like gender and age may facilitate or deter workaholism. According to the present study findings, males are more workholics than females. This agrees with Burke and Koksal [48], and could be demonstrated by tendency of males to work longer hours (53). The reverse was found by Buelens and Poelmans (28) while Porter (50) failed to find a difference. In the present study, workaholism correlated with younger age group and this could be explained by the wise up experienced at old ages besides adjustment the work pattern due to being committed (e.g., having a family). This agrees with Andreassen et al. (54) and Taris et al. (55).

### Relation Between Workaholism and Burnout and Inflammatory Markers

The present results showed that working excessively was a predictor to burnout (Emotional exhaustion and depersonalization) and this agrees with Nonnis et al. (2). It seems that workaholics over time are more vulnerable to quit working to a higher degree than non-workaholics. On studying burnout among HCWs with critical specialty, the results revealed that burnout is a strong predictor to pro-inflammatory cytokines TNF-α, IL6, and CoQ10. This agrees with some studies (16, 56, 57). Through their action on the brain, they cause behavioral symptoms of sickness like fatigue, sleepiness, loss of appetite and decreased libido (57). On the other hand, the enzymatic antioxidants level (co-enzyme Q10) decreased in depressed patients (58, 59). Recently researches prove that lower CoQ10 plays a role in the pathophysiology of depression. It is suggested that depressed patients may benefit from CoQ10 supplementation (58).

## Strengths and Limitations

The present study was conducted in Egypt, thus the findings cannot necessary be generalized to workers from other countries. The work did not focus on the type of work whether organizationally employed or self-employed giving some limits on the possible covariates that may be associated with work addiction. The study was a cross-sectional one, so no conclusions can be drawn in terms of cause-and-effect relationships between study variables. Furthermore, all data were based on self-report and there is no gold standard for assessment of workaholism so this is a point of debate (60). Despite these limitations, there are points of strength. The work is (to the best of the authors' knowledge) the first to assess workaholism in a representative sample of workers in Egypt. The study included questionnaires and laboratory investigations to assess the association between work addiction and mental health in addition to burnout. As noted, the response rate (96.60%) is so acceptable. The study included 1,080 participants which provides high statistical power and consequently decreases the probability of Type 2 errors (61).

## Conclusion

There is a significant association between workaholism and psychologically poor-health and poor quality of life among HCWs. Critical specialty healthcare workers showed association between workaholism, burnout, and pro-inflammatory markers. Addressing of personal characteristics, supporting factors in the work environment and periodic examination of the healthcare workers and responding accordingly is required.

## Data Availability Statement

The raw data supporting the conclusions of this article will be made available by the authors, without undue reservation.

## Ethics Statement

The studies involving human participants were reviewed and approved by Institutional research board at Menoufia Faculty of Medicine, Egypt. The patients/participants provided their written informed consent to participate in this study.

## Author Contributions

All authors certify that they have participated sufficiently in the work to take public responsibility for the content, including participation in the concept, design, analysis, writing, or revision of the manuscript. ZK had the role of getting the idea, performing statistical analysis, writing the methodology and results sections, final revision, and publishing. ME had the role of writing the manuscript (Introduction) and conducting revision. EA-E, AAb, NB, HAb, SS, SA, AHA, ARA, HAm, NA, and AR collected data and share in writing (Methods: choice of tools and Discussion) and revision of the manuscript. AAl conducted the revision and editing.

## Conflict of Interest

The authors declare that the research was conducted in the absence of any commercial or financial relationships that could be construed as a potential conflict of interest.
